# Detecting the Cool Island Effect of Urban Parks in Wuhan: A City on Rivers

**DOI:** 10.3390/ijerph18010132

**Published:** 2020-12-27

**Authors:** Qijiao Xie, Jing Li

**Affiliations:** 1 Faculty of Resources and Environmental Science, Hubei University, Wuhan 430062, China; 17865818365@163.com; 2 Key Laboratory of Regional Development and Environmental Response, Wuhan 430062, China

**Keywords:** park cool island, blue-green space, water body, park design, nature-based solution

## Abstract

As a nature-based solution, development of urban blue-green spaces is widely accepted for mitigating the urban heat island (UHI) effect. It is of great significance to determine the main driving factors of the park cool island (PCI) effect for optimizing park layout and achieving a maximum cooling benefit of urban parks. However, there have been obviously controversial conclusions in previous studies due to varied case contexts. This study was conducted in Wuhan, a city with high water coverage, which has significant differences in context with the previous case cities. The PCI intensity and its correlation with park characteristics were investigated based on remote sensing data. The results indicated that 36 out of 40 urban parks expressed a PCI effect, with a PCI intensity of 0.08~7.29 °C. As expected, larger parks with enough width had stronger PCI intensity. An increased density of hardened elements in a park could significantly weaken PCI effect. Noticeably, in this study, water bodies in a park contributed the most to the PCI effect of urban parks, while the vegetated areas showed a negative impact on the PCI intensity. It implied that in a context with higher water coverage, the cooling effect of vegetation was weakened or even masked by water bodies, due to the interaction effect of different variables on PCI intensity.

## 1. Introduction

Worldwide, the urban heat island (UHI) effect has been detected in megacities and also in small towns in recent years [1,2]. The UHI effect usually leads to a series of adverse economic and environmental consequences [3,4]. Higher temperatures in urban areas increase the energy consumption for building cooling and influences the energy layout [5,6], contributing much to global warming [7]. More anthropogenic heat and pollutants are released into the atmosphere from industries and energy plants, which intensifies the UHI effect and raises the air pollution level in urban areas [8]. As reported, the air pollutant concentration in urban areas was approximately 10 times that in a clean environment [9]. The superposition of heat stress and air pollution in urban areas may exacerbate the negative impacts on an urban residential environment [10,11]. The worsening thermal environment significantly leads to high morbidity and mortality of the heat-related diseases, especially in extreme heat events [4,12]. As a result, how to mitigate the UHI effect has been extensively discussed in many studies [3,13,14,15,16].

As a nature-based solution, the development of blue-green spaces in urban areas has been widely recommended to mitigate the UHI effect due to the high cost-effectiveness and environmental friendliness [4,16,17,18,19,20]. Urban vegetation can decrease temperature by shading and by absorbing radiation energy through photosynthesis and transpiration [21]. Water bodies cool the environment by transforming sensible heat to latent heat through water evaporation [22,23]. In addition, due to the high heat capacity and enthalpy of vaporization, water bodies act as thermal buffers in urbanized environments, which moderate temperature variation [24]. Urban parks, generally containing both vegetation and water bodies, are usually cooler than their surrounding urbanized areas and create a park cool island (PCI) effect [21,25,26,27,28]. 

There have been significant differences in PCI values among different studies. For example, the mean PCI intensity of the urban parks in a highly urbanized region in Beijing, China was 0.68 °C (−4.61~4.65 °C), with 48 of 266 parks not showing PCI effect [25], while the mean PCI values for 18 parks in another Chinese city, Changzhou was 3.65 °C [26]. The average PCI values for 92 parks in Nagoya, Japan were 1.30 K (0.40~4.20 K) in summer, 1.16 K (0.51~4.14 K) in spring, and 0.43 K (−0.45~1.84 K) in autumn [27]. The 61 parks in Taipei were detected to be, on average, 0.81 K cooler than the surroundings, with the PCI intensity ranging from 0.16 K to 0.57 K in different observed times [21]. Generally, the PCI intensity varied with the location, size, shape, composition, and configuration of the sample parks [4,16,29]. Although different studies had different conclusions on the main factors affecting the PCI intensity of urban parks [13,16,29], it was popularly acknowledged that PCI effect was significantly correlated with park area [16,30]. Large urban parks usually experience much lower temperatures than the surrounding environment. In contrast, those small-sized parks show weaker PCI effect or even a UHI effect, especially in highly urbanized areas [26,27]. It implies that the larger the park area, the stronger the PCI effect [13]. On the basis of these findings, large-sized parks are considered to be an optimal solution for achieving the maximum cooling benefit. The fact is, urban park area cannot be expanded limitlessly due to the urbanization and economic development [13,31]. 

Therefore, how to maximize the PCI intensity of a park is of great significance as the park area is given. Much attention has been paid to the relationship between PCI intensity and park characteristics, which is needed for urban planning and park design [4,13,16,31]. Tree canopy coverage, leaf area index, water cover, and impervious surface area have been considered to be the main driving factors for the formation of PCI effect [3,29], while the contribution of different park characteristics to the PCI intensity of the park remains uncertain [13]. For example, Cao et al. (2010) considered the vegetation coverage and shape index as the dominant indicators impacting the park cooling effect [27]. Qiu and Jia (2020) claimed that expanding tree canopy and limiting impervious surface could intensify the PCI effect [25]. Nevertheless, other researchers reported that water bodies contributed more to the PCI intensity of a park than urban vegetation [13,32]. The PCI intensity can be higher with the water proportion in a park exceeding 30% [33]. The controversial conclusions can be explained by the differences in urban roughness and landscape context among varied case cities [2,13]. However, most of the previous studies were conducted in cities with low water coverage, which might underestimate the contribution of water bodies to the PCI effect [27]. 

This study was conducted in Wuhan, China. Wuhan is known as the “city with hundreds of lakes” due to the high-water coverage and a large number of water bodies. About 72.5% of the sample parks contain water bodies, with the percentage of water area in parks ranging from 2.35% to 82.92%. The aims of this study are the following: (1) to examine the PCI effect of the municipal comprehensive city parks, (2) to quantify the PCI intensity in parks of different sizes, (3) to detect the correlations among PCI intensity and park characteristics, and (4) to investigate the dominant impact factors of urban parks on the cooling effect in a water-rich context. The results can help urban planners and park designers to further understand how park characteristics influence PCI effect in different contexts and provide invaluable practical advice for urban planning and park structure optimization with consideration of water bodies.

## 2. Materials and Methods 

### 2.1. Study Area and Park Samples

Wuhan (113°41′~115°05′ E, 29°58′~31°22′ N), the capital of Hubei province, is one of the largest cities in China. It is located in the north-subtropical climatic zone with four distinct seasons. It has cold winters and hot summers with sufficient heat and abundant rainfall. The average annual temperature is 15.8 to 17.5 °C and the average annual rainfall is 1150 to 1450 mm, of which 40% is concentrated during June to August. Wuhan has an area of more than 8569 km^2^ with a built-up area of 812.4 km^2^. The Yangtze River and the Han River meet in the center of Wuhan and divide the city into three parts. The crisscross water network with many rivers, lakes, and interwoven reservoirs has a total area of 2217.6 km^2^, accounting for 26.1% of the city’s area. 

**Figure 1:**
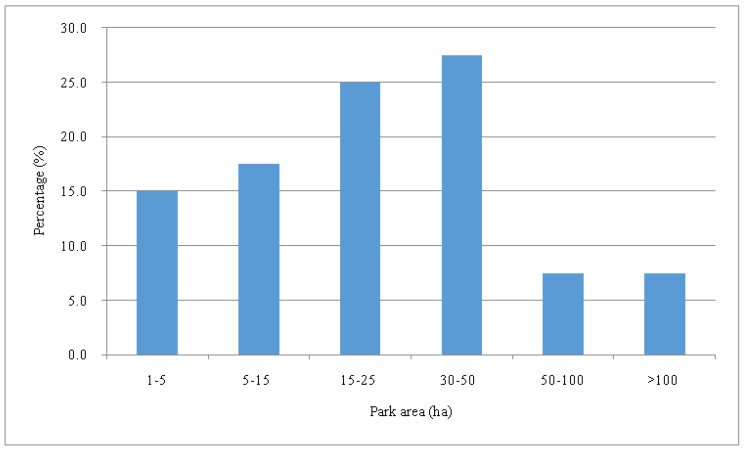
Number proportion of urban parks with different sizes.

There are 57 urban parks in the Wuhan urbanized area. Considering the representativeness of the sample parks, those with special land covers and surface characteristics were not included. Forty municipal comprehensive city parks were selected as the samples with forest parks and those dominated by hard pavement were excluded in this study. The mean area of the sample parks was 35.8 ha. The smallest park was Shuiguohu Park (SGH) with an area of 1.4 ha and the largest one was Shahu Park (SH) of 324.6 ha. Figure 1 shows the number proportion of the sample parks with different sizes. There were 21 parks with an area range of 15–50 ha, accounting for 52.5% of all parks. There were 6 small-sized (1–5 ha) and 6 large-sized (larger than 50 ha) parks, with the total proportion of 15%.

### 2.2. Park Structure Characteristics

The boundaries of the sample parks were detected and vectorized based on the Google Earth image. For each park, combined with the necessary field confirmation, a maximum likelihood method was performed in Envi 5.2 to divide the land use/land cover (LULC) into four types (woodland, lawn, water body, and hard pavement). The classification accuracy was verified with a value of more than 85%. A total of 24 indexes were selected to indicate park characteristics. Four descriptors, namely, size/area (S), circumference (C), width (W), and shape index (SI), were used to indicate the geometry of each park. Twelve common indexes such as the normalized difference vegetation index (NDVI), normalized difference built-up index(NDBI), modified normalized difference water index (MNDWI), vegetation coverage (VC), as well as area (A_i_) and proportion (P_i_) of different landscape types (i refers to woodland, lawn, water body, and hard pavement) were selected to indicate the inner composition of each park. Eight typical indices including the shape index (SI_i_) and contagion index (CI_i_) of the four land-cover types were used to represent the spatial configuration in each park. The indexes of SI, NDVI, NDBI, MNDWI, and CI were calculated as follows:

(1) $$\mathrm{SI}=C/(2*\sqrt{\pi *A})$$

where *C* and *A* are the circumference and area of a park or different land cover, respectively.

CI = 1 − C_o_/C_omax_ (2)

where CI is the contagion index, C_o_ is the complexity index, and C_omax_ is the maximum value of C_o_.

NDVI = (*ρ*(NIR) − *ρ*(Red))/(*ρ*(NIR) + *ρ*(Red)) (3)

NDBI = (*ρ*(MIR) − *ρ*(NIR))/(*ρ*(MIR) + *ρ*(NIR)) (4)

MNDWI = (*ρ*(Green) − *ρ*(MIR))/(*ρ*(Green) + *ρ*(MIR)) (5)

where *ρ*(NIR), *ρ*(Red), *ρ*(MIR), and *ρ*(Green) are the reflectance values of near-infrared band, of red band, of mid-infrared band, and of green band of Landsat 8, respectively [34,35].

### 2.3. LST Derivation

In this study, a method of single-channel algorithm was used to derive the land surface temperature (LST) in Envi 5.2 based on the Landsat 8 image with cloud coverage of 3.11%, acquired on 17 August 2018. After the radiometric and atmospheric correction, the spectral radiance and TOA (top of the atmosphere) reflectance for each band were obtained [34,35]. Then, the LST values can be calculated based on TIRS 10 (with a spatial resolution of 100 × 100 m) of Landsat 8 image by the formulas:

(6) $$LST=\{a(1-c-d)+[b(1-c-d)+c+d]T_{i}-d*T_{a}\}/c$$

where *a* and *b* are constants with the values of −67.355351 and 0.4558606, respectively; *T_a_* is the TOA values; *T_i_* is the brightness temperature which can be obtained from Equation (7); *c* and *d* are intermediate variables from Equations (8) and (9) as follows:

*T_i_* = *K*_2_/ln [*K*_1_/*L_λ_* + 1] (7)

where *L_λ_* is the spectral radiance, *K*_1_ = 774.89 W/(m^2^μmsr) and *K*_2_ = 1321.08 K.

(8) $$c=\varepsilon_{i}*\tau_{i}$$

(9) $$d=(1-\varepsilon_{i})[1+(1-\varepsilon_{i})*\tau ]$$

where $\varepsilon_{i}$ is the land surface emissivity correlated with the NDVI index [36,37]; τ is atmospheric transmissivity, which is calculated using NASA’s atmospheric correction parameter calculator [38].

### 2.4. Cool Island Intensity Identification

Following the definition of UHI intensity as temperature difference between urban areas and the surrounding suburbs [39], the PCI effect was described as the phenomenon that urban parks experience lower temperatures than the urbanized areas [27,28]. In this study, the PCI intensity was defined as the LST difference between inside and outside of a park based on the following equation:

PCI = T_o_ − T_p_ (10)

where PCI is the value of the PCI intensity for a park, T_o_ is the average LST value outside a park in an urbanized context with blue-green spaces excluded, and T_p_ is the mean LST value inside the park.

### 2.5. Data Analysis

Previous studies have confirmed that the LST value is context sensitive and easily impacted by the local microclimate [3,28]. To eliminate the influence of the surrounding environment on the relationship between PCI intensity and park characteristics, the parks close to large water bodies were excluded when illustrating the relationships. For each of the remained 27 sample parks, the values of PCI and the variables such as S, C, W, NDVI, NDBI, MNDWI, VC, A_wa._, A_la._, A_wo._, A_ha._, P_wa._, P_la._, P_wo._, and P_ha._ were counted in ArcGIS 10.2. The other indexes, namely SI, CI_wa._, CI_la._, CI_wo._, CI_ha._, SI_wa._, SI_la._, SI_wo._, and S_ha._ were calculated in Fragstas 4.2 based on the LULC classification. Then, the mean PCI intensity of the sample parks and the corresponding variables associated with park characteristics were collected in an Excel database. The correlation analysis between 24 selected indexes and the mean PCI values were performed in SPSS 22. To examine the main impact factors and their influencing mechanism on PCI values, a multiple step wise regression method was also carried out.

## 3. Results

### 3.1. Cool Island Effect of Urban Parks

**Figure 2:**
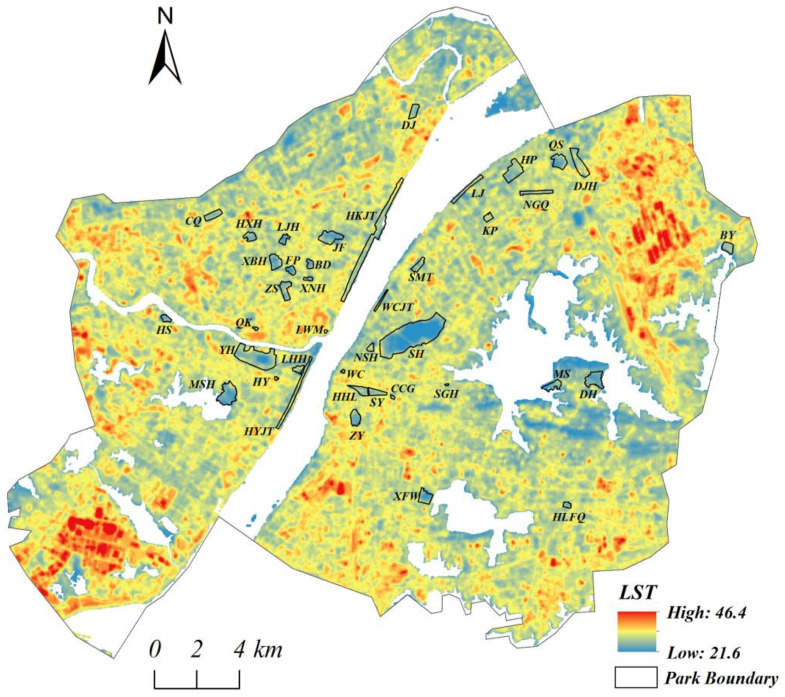
Spatial distribution of LST (land surface temperature, °C) (water bodies outside eliminated).

Figure 2 displays the spatial distribution map of LST values with the water bodies outside the sample parks eliminated. The boundaries of 40 sample parks are marked with a black line. High temperatures were mainly distributed in the built-up areas, especially in the industrial areas. Compared with the surrounding urbanized areas, urban parks expressed an obvious “cool island effect” with lower LST values inside the park. Table 1 lists the average LST values and the standard deviation (SD) values in different contexts. The mean LST, within the sample parks, was 29.31 °C, which was lower than the average LST of the whole study area (water bodies excluded) (30.88 °C) and of that in the built-up area (blue-green areas excluded) (31.54 °C). However, it was higher than the average LST value of the blue-green areas, by 1.5 °C. The SD values of the LST within the sample parks were much lower than those in the other three contexts, indicating a relatively narrow variation range of LST values in urban parks as compared with the total study area with water bodies excluded, which had the largest SD value, due to the highest spatial heterogeneity. 

### 3.2. Cool Island Intensity

Table 2 shows the mean LST and PCI values for different sample parks. The average LST values of different parks ranged from 24.25 to 31.98 °C. The majority of the sample parks experienced lower temperatures than the surrounding areas, with the PCI values of 0.08~7.29 °C, a wide variation range. However, four parks, namely, the Nanganqu (NGQ) Park, the Wuchang (WC) Park, the Longwangmiao (LWM), and the Changchunguan (CCG) Park had higher temperatures than their surroundings by 0.10 °C to 0.44 °C. Out of these four parks, three were less than 5 ha in areas. 

**Figure 3:**
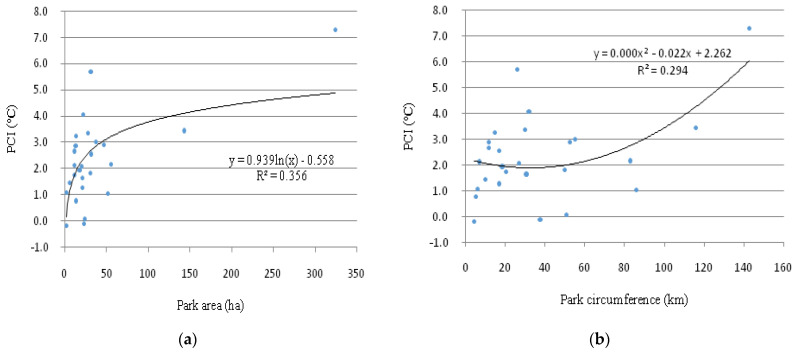
Scatterplots and fitting curves between the PCI intensity and (**a**) park area at the significance level of 0.01 and (**b**) park circumference at the significance level of 0.05 (*n* =27).

Figure 3 illustrates the correlations of PCI intensity with the area (S) (Figure 3a) and circumference (C) values (Figure 3b) of urban parks. With the increase of park area and perimeter, the PCI value tends to increase correspondingly, suggesting that park size and circumference were the contributors to PCI effect. However, the correlation between PCI and park geometry was nonlinear. The determination coefficients (R^2^) of the regression equations between PCI and park size, as well as park circumference were 0.356 and 0.294, respectively. It meant that park size and perimeter alone could not efficiently explain the PCI variation.

### 3.3. Correlation between PCI and Park Characteristics

Table 3 indicates the correlation coefficients between PCI intensity and 24 variables related to park characteristics. The PCI intensity was significantly correlated with the park size/area (S), area of water bodies (A_wa._), area proportion of water bodies (P_wa._), area proportion of woodland (P_wo._), NDBI and MNDWI values at the significance level of 0.01. It was also significantly correlated with park circumference (C), park width (W), area proportion of hard pavement (P_ha._), vegetation coverage (VC), the shape index of woodland (SI_wo._), and NDVI value at the significance level of 0.05. There were negative correlations between the PCI intensity and the indexes of NDBI and P_ha._, as expected. The cooling effect was weakened by the increasing proportion of hard pavement. The geometry of urban parks (S, C, and W) was positively correlated to the PCI intensity, which meant that in addition to expanding a park, increasing the circumference and width of a park as the park area was fixed also helped to improve the PCI effect. Water bodies significantly contributed to the cooling effect of urban parks with all water-related variables (A_wa._, P_wa._, and MNDWI) positively correlated to the PCI intensity. Noticeably, in this study, negative correlation existed between PCI intensity and P_wo._, VC, and SI_wo._, suggesting that vegetation negatively influenced the cooling effect of urban parks. This was partly attributed to the large proportion of water bodies inside the sample parks and the dense and high buildings outside the parks. 

Table 4 shows the multiple stepwise regression models between the PCI intensity and the park characteristics. When the interaction of all variables influencing the PCI intensity was considered, only NDBI and P_wa._ remained as the main impact factors. These two indicators combined to explain 87.9% of the PCI variation. The standardized regression equation provided the information that the NDBI contributed much more to the cooling effect of urban parks than P_wa_. It meant that the PCI intensity was more sensitive to the increase of impervious areas. When the other variables remained unchanged, the PCI intensity was decreased by 1.8 °C for each 0.1 increase in the NDBI. The PCI intensity was increased by about 0.5 °C for each 10% increase in area proportion of water bodies.

## 4. Discussion

### 4.1. Park Geometry and PCI Intensity

Urban parks usually express an obvious cool island effect due to the concentrated blue-green spaces in urban areas [13,40].The PCI effect was detected in most of the sample parks in our study. However, there were still four parks that showed a UHI effect with the LST values higher than the surroundings (Table 2). This finding was consistent with the previous studies conducted in Taipei and Leipzig [21,41]. Park geometry such as park size, park shape and park circumference were closely correlated to the PCI intensity [21,32,40,42,43].

In this study, the PCI intensity was significantly correlated with park size (*p* < 0.01), park circumference, and park width (*p* < 0.05) but the correlations were nonlinear (Figure 3 and Table 3). A large park (usually has large circumference) represents higher natural coverage, greater green biomass, and larger shading area, which are beneficial to the cooling effect by photosynthesis and transpiration process [16,30,44]. Additionally, a larger park implies a stable microclimate condition and is less affected by the warmer environment around it [28]. Correspondingly, a small park is sensitive to the thermal environment, which has a negative impact on the cooling effect [41]. It was confirmed by our finding that the UHI effect or weaker PCI intensity was detected in smaller parks with an area less than 5 ha (Table 2). Similarly, the parks with a smaller width were more vulnerable to the influence of the surrounding environment than those with larger width [21], which meant that the belt-shape parks tended to have low PCI intensity or even express UHI effect [27]. These could explain why some medium or large parks such as the Nanganqu (NGQ) Park, the Linjiang Park (LJ), and the Hankoujiangtan (HKJT) Park have relatively higher temperatures (Table 2 and Figure 3). 

### 4.2. Park Characteristics and PCI Intensity

**Figure A1:**
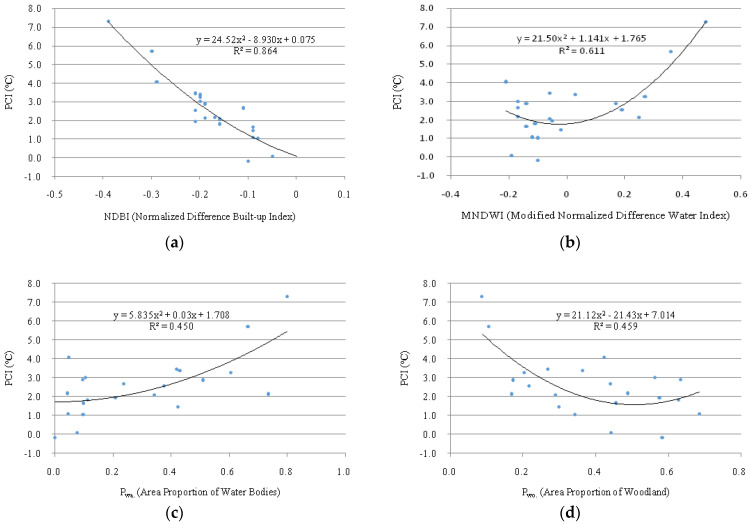

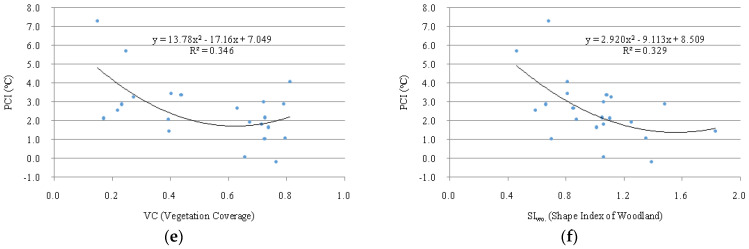
Correlations among PCI and the main variables. (**a**) NDBI (normalized difference built-up index), (**b**) MNDWI (modified normalized difference water index), (**c**) P_wa._ (area proportion of water bodies), (**d**) P_wo._ (area proportion of woodland), (**e**) VC (vegetation coverage), and (**f**) SI_wo._ (shape index of woodland).

A wide variation range of PCI intensity (0.088–7.298 °C) was detected in this study (Table 2). In addition to park geometry, some other impact factors that might explain the PCI variation were further investigated based on the correlation analysis (Table 3). The PCI intensity was significantly correlated to the NDBI (*p* < 0.01) and the area proportion of hardened surfaces (P_ha._) in a park (*p* < 0.05), with the correlation coefficient of −0.920 and −0.490. The NDBI was usually developed for identifying the built-up areas or construction density [45], which indicated the hardening degree of urban parks. Impervious surfaces in parks absorb more solar radiation and consequently increase the LST values, which negatively influence the cooling effect of urban parks [25,28,46]. As shown in Figure A1a, the NDBI alone could explain 86.4% of the PCI variation.

The PCI intensity was positively and significantly correlated to A_wa._, P_wa_, and MNDWI at the significance level of 0.01 (Table 3 and Figure A1b,c). As Lin et al. (2015) and Yu (2018) reported, the cooling intensity of water bodies was stronger than that of the vegetated areas [13,32]. The larger the proportion of water in a park accounted for, the stronger the cooling effect of the park was [33]. However, not all researchers believed that water cover was efficient in cooling the surroundings. Small water bodies, especially in dense urban areas might play an insignificant or negative role in impacting the PCI intensity [22,27]. These controversial results could be interpreted by different backgrounds or contexts of specific studied cities [13,19,28]. For example, Cao et al. (2010) found that water bodies in urban parks played an unimportant role in PCI effect in Nagoya, Japan [27]. They considered that this result was mainly attributed to the low frequency and small area of the water bodies. In the present study, more than 70% of the sample parks contained water bodies and the water area covered 34.8% of the total areas of all sample parks. Combined with the other blue-green spaces outside the sample parks, they formed a stable network of open spaces, which improved the PCI effect of urban parks.

It is noteworthy that the PCI intensity was significantly but negatively correlated to P_wo._, SI_wo_, and VC (Figure A1d–f). The area and shape of woodland were found to negatively impact the cooling effect of urban parks. This finding was contrary to most of the previous studies, which concluded that the PCI intensity was mainly influenced by the area of trees and shrubs in a park [13,21,27,28,42]. However, in their studies, several parks with higher tree/shrub coverage, especially those in highly developed areas, were detected to experience higher temperatures and to express a heat island effect. The environmental characteristics around the tree/shrub or vegetation can affect the cooling effect of urban parks [13,19,25,28,31], suggesting that the PCI effect was context sensitive. This phenomenon was mainly reported in highly urbanized environments [3,4,16]. Our results confirmed that it also existed in a context with higher water coverage. Water bodies could influence the cooling efficiency of urban vegetation on the PCI effect, which was partly attributed to the interaction effects in different variables. As Table A1 shows, P_wo._ and VC are significantly correlated to A_wa._, P_wa._ and MNDWI.

### 4.3. Park Design and the PCI Effect Improvement

Traditionally, urban parks were mainly planned and built for recreation and entertainment. Currently and in the future, urban parks are additionally planned to adapt to climate change and fight against negative environmental problems [13,16,42]. In terms of the impact of urban parks on the thermal environment, a large-sized park is optimal due to the open spaces and significant green biomass [13,26,31]. However, belt-shape parks were easily influenced by the surrounding warmer environment, which weakened the PCI effect, although their areas were not small (Figure 2). Larger parks with enough width were more efficient in improving the PCI effect [21]. However, it is unrealistic to improve the cooling efficiency by increasing the park area without restriction [13,23,31].

Given a fixed park size, the PCI intensity generally depends on the park characteristics including both landscape composition and configuration [26,28,31,47,48]. In this study, the PCI intensity had a much closer correlation with the variables indicating landscape composition such as A_wa._, P_wa._, P_wo._, NDBI, and MNDWI than with those indicating landscape configuration (Table 3). It suggested that appropriate proportion of different land cover types in a park was important in maximizing the cooling effect of urban parks.

The NDBI and proportion of water area (P_wa._) were considered to be the dominant factors for impacting the cooling effect of sample parks taking into consideration the interaction effects of different variables on the PCI intensity (Table 4). Increased NDBI values could significantly weaken the cooling effect of urban parks [28,46]. In urban open parks, the NDBI value was closely related to the density of roads, pavement, squares, and parking lots [4,19]. Controlling the proportion of hardened surface and increasing the natural surface area are important measures to improve the cooling effect of a park during the process of park planning. Using alternative cool paving materials to replace impervious surfaces was another solution when the infrastructure construction in a park is inevitable [49].

Water bodies were the main positive contributors to the PCI intensity (Table 4). Urban planners and managers have considered water bodies as vital elements to mitigate UHI intensity [19,50]. It has been reported that water bodies had an average cool intensity of 2.5 K [20]. In addition, the cooling effect can be improved with enlarged size of water bodies [19,33]. Consistent with these conclusions, the water body area was found to significantly influence the PCI intensity in this study (Table 3 and Figure A1d). Protecting urban water bodies, especially the larger ones, is of great significance for improving thermal comfort [20,22,23]. Nevertheless, accelerating urbanization leads to water area reduction and fragmented landscape of water network in urban areas, which consequently deteriorates the surrounding thermal conditions [3,11,50]. Connecting small-sized water bodies and building ventilation channels to restore a water network can provide an efficient cooling effect on thermal environment in dense built-up areas [23]. 

### 4.4. Limitations and Prospects

This study investigated the impact of urban parks on PCI intensity in the perspective of park design. The emphasis was placed on the influence of the internal characteristics of sample parks themselves, without considering the external impacting factors. However, according to the definition of the PCI effect, it is the combined result of the driving factors, both inside and outside urban parks [3,25,28]. The surrounding environmental conditions also contribute to PCI variation, which may partly influence our results. Our study confirmed that park area was one of the main factors that influenced the PCI effect of urban parks (Table 3), while the park area alone could not efficiently explain the variation of PCI intensity (Figure 3a). As suggested, the PCI intensity of urban parks might have one or more thresholds in park size and the impact of urban parks on PCI effect might be discussed at different thresholds [13]. In addition, this study was conducted in a city with greater water coverage, which, to some extent, extended the study samples in different contexts. The degree to which significant differences between our study and some previous studies should be attributed to context variation needs to be further discussed. 

## 5. Conclusions

This study quantified the PCI effect of the municipal comprehensive parks in the urbanized area in Wuhan, a “city with hundreds of lakes”. About 90% of the tested parks expressed a cool island effect, with the PCI values of 0.08 to 7.29 °C. Larger parks with enough width were more efficient for improving the PCI effect. Those parks with small size or in a belt shape expressed a weak PCI effect, and even a UHI effect. The PCI intensity was negatively correlated to the indexes of NDBI and P_ha._, as expected. With the density increase of roads, pavement, squares, and parking lots in urban parks, the PCI intensity decreased significantly. Water bodies were the main contributors to the PCI effect, with the PCI intensity positively correlated to A_wa._, P_wa_, and MNDWI. In this study, the PCI intensity of water bodies was much stronger than that of vegetated areas, which was mostly attributed to the high coverage of water bodies, both in the sample parks and in an urban context. The PCI intensity was negatively correlated to P_wo._, SI_wo_, and VC. In a context with greater water coverage, the cooling effect of vegetation was weakened or even masked by water bodies, due to the interaction effect of different variables on the PCI intensity. In terms of park design to improve the PCI effect, it was efficient by increasing park area, protecting large water bodies in parks, controlling hardened surface density, and using alternative cool paving materials.

## Figures and Tables

**Table 1:** Statistics of mean LST (land surface temperature) and SD (standard deviation) values.

Context	Mean LST (°C)	SD (°C)
Whole area (water bodies excluded)	30.88	3.25
Built-up area (blue-green areas excluded)	31.54	3.02
Blue-green area	27.81	2.06
Considered urban parks	29.31	1.77

**Table 2:** Basic information and park cool island (PCI) intensity for different sample parks.

No.	Park	Full Name of Park	Area (ha)	Perimeter (km)	PCI (°C)	T_mean_ (°C)	SD (°C)
1	ZS	Zhongshan Park	30.49	49.64	1.82	29.72	1.45
2	XNH	Xiaonanhu Park	6.05	9.90	1.46	30.08	1.88
3	SH	Shahu Park	324.62	142.66	7.29	24.25	3.63
4	LWM	Longwangmiao Park	1.92	4.83	−0.24	31.78	1.15
5	HY	Hanyang Park	2.23	5.73	1.08	30.46	1.17
6	HKJT	Hankoujiangtan Park	147.13	135.21	0.91	30.63	1.53
7	CQ	Changqing Park	23.86	50.43	0.08	31.46	1.38
8	BD	Baodao Park	11.29	6.75	2.14	29.4	2.43
9	DWY	Dongwuyuan Park	67.31	54.73	3.96	27.58	2.31
10	ZWY	Zhiwuyuan Park	47.07	64.49	5.15	26.39	1.66
11	QK	Qiaokou Park	2.89	4.46	0.59	30.95	1.34
12	ZY	Ziyang Park	27.97	29.93	3.37	28.17	2.1
13	BY	Baiyu Park	21.8	31.69	4.07	27.47	2.14
14	CCG	Changchunguan Park	2.56	2.81	−0.44	31.98	0.94
15	SGH	Shuiguohu Park	1.38	1.48	0.22	31.32	0.87
16	WC	Wuchang Park	2.13	4.17	−0.17	31.71	1.02
17	NSH	Neishahu Park	8.88	8.27	2.78	28.76	1.89
18	HS	Hanshui Park	11.3	11.61	2.67	28.87	1.4
19	DJ	Dijiao Park	20.92	30.41	1.66	29.88	1.77
20	KP	Kepu Park	11.63	20.31	1.76	29.78	1.39
21	JF	Jiefang Park	46.78	52.26	2.9	28.64	1.62
22	HP	Heping Park	55.47	82.68	2.17	29.37	1.86
23	PQ	Penquan Park	13.17	4.95	0.77	30.77	1.95
24	QS	Qingshan Park	37.22	54.92	3.01	28.53	1.71
25	LJH	Lingjiaohu Park	13.45	14.46	3.26	28.28	2.67
26	LHH	Lianhuahu Park	13.06	11.39	2.88	28.66	2.5
27	HXH	Houxianghe Park	17.74	18.22	1.95	29.59	1.58
28	DHMY	Donghumeiyuan Park	24.71	38.01	3.63	27.91	2.68
29	HLFQ	Helanfengqing Park	8.37	16.02	0.58	30.96	1.43
30	HYJT	Hanyangjiangtan Park	46.88	61.40	1.94	29.6	1.61
31	WCJT	Wuchangjiangtan Park	8.79	17.48	3.13	28.41	1.69
32	SMT	Simeitang Park	19.93	26.81	2.08	29.46	2.1
33	LJ	Linjiang Park	27.93	40.02	4.82	26.72	2.14
34	YH	Yuehu Park	143.47	115.61	3.45	28.09	2.65
35	HHL	Huanghelou Park	22.51	23.34	0.49	31.05	1.29
36	SY	Shouyi Park	20.83	16.89	1.29	30.25	1.42
37	DJH	Daijiahu Park	51.91	85.97	1.04	30.5	1.8
38	XFW	Xingfuwan Park	31.23	25.88	5.7	25.84	3.28
39	XBH	Xibeihu Park	31.36	16.82	2.55	28.99	2.93
40	NGQ	Nanganqu Park	22.7	37.36	−0.1	31.64	1.36

All the sample parks are transliterated in full names. LST, land surface temperature; PCI, park cool island; T_mean_, the mean value of land surface temperature; SD, standard deviation.

**Table 3:** Correlation coefficients between PCI and park structure characteristics (*n* =27).

Index	Description	Coefficient	Index	Description	Coefficient
S	Park size/area	0.664 **	VC	Vegetation coverage	−0.519 *
C	Park circumference	0.439 *	CI_wa._	Contagion index of water bodies	0.403
W	Park width	0.512 *	CI_la._	Contagion index of lawn	0.11
SI	Shape index	0.05	CI_wo._	Contagion index of woodland	−0.178
A_wa._	Area of water bodies	0.693 **	CI_ha._	Contagion index of hard pavement	0.026
A_la_.	Area of lawn	0.37	SI_wa._	Shape index of water bodies	0.057
A_wo._	Area of woodland	0.306	SI_la._	Shape index of lawn	0.228
A_ha_.	Area of hard pavement	0.357	SI_wo._	Shape index of woodland	−0.519 *
P_wa._	Area proportion of water bodies	0.646 **	SI_ha._	Shape index of hard pavement	0.389
P_la_.	Area proportion of lawn	−0.19	NDVI	Normalized difference vegetation index	−0.480 *
P_wo._	Area proportion of woodland	−0.567 **	NDBI	Normalized difference built-up index	−0.920 **
P_ha_.	Area proportion of hard pavement	−0.490 *	MNDWI	Modified normalized difference water index	0.645 **

** Correlation is significant at the 0.01 level 2-tailed; * Correlation is significant at the 0.05 level 2-tailed.

**Table 4:** Multiple stepwise regression models between PCI and park structure characteristics at the significance level of 0.01 (*n* =27).

PCI Intensity	Regression Equation	R^2^
Standardized regression equation	$Y=0.185\times P_{\mathrm{wa}.}-0.874\times \mathrm{NDBI}$	0.879 **
General regression equation	$Y=4.718\times P_{\mathrm{wa}.}-17.997\times \mathrm{NDBI}-1.979$	

** Correlation is significant at the 0.01 level 2-tailed. PCI, park cool island; P_wa._, area proportion of water bodies; NDBI, normalized difference built-up index.

## Data Availability

Data sharing not applicable.

## Abbreviations

UHI	Urban heat island
PCI	Park cool island
LULC	Land use/land cover
LST	Land surfacetemperature
SD	Standard deviation
NDVI	Normalized difference vegetation index
NDBI	Normalized difference built-up index
MNDWI	Modified normalized difference water index
S	Size/area
C	Circumference
W	Width
SI	Shape index
A_wa._	Area of water bodies
A_la_.	Area of lawn
A_wo._	Area of woodland
A_ha_.	Area of hard pavement
P_wa._	Area proportion of waterbodies
P_la_.	Area proportion of lawn
P_wo._	Area proportion of woodland
P_ha_.	Area proportion of hard pavement
VC	Vegetation coverage
CI_wa._	Contagion index of water bodies
CI_la._	Contagion index of lawn
CI_wo._	Contagion index of woodland
CI_ha._	Contagion index of hard pavement
SI_wa._	Shape index of water bodies
SI_la._	Shape index of lawn
SI_wo._	Shape index of woodland
SI_ha._	Shape index of hard pavement

## Appendix A

**Table A1:** Pearson correlationsamongthe PCIvalues and all variables indicating park structure characteristics.

	PCI	S	C	W	SI	A_wa._	P_wa._	A_wo._	P_wo._	A_gr._	P_gr._	A_ha._	P_ha._	VC	CI_wa._	CI_wo._	CI_gr._	CI_ha._	SI_wa._	SI_wo._	SI_gr._	SI_ha._	NDVI	NDBI
S	0.664 **																							
C	0.439 *	0.855 **																						
W	0.512 *	0.710 **	0.901 **																					
SI	0.05	0.077	0.039	0.045																				
A_wa._	0.693 **	0.963 **	0.696 **	0.523 *	0.103																			
P_wa._	0.646 **	0.411	0.086	0.16	0.394	0.526 *																		
A_wo._	0.306	0.645 **	0.881 **	0.851 **	−0.11	0.429 *	−0.125																	
P_wo._	−0.567 **	−0.373	−0.159	−0.282	−0.461 *	−0.445 *	−0.874 **	0.132																
A_gr._	0.37	0.737 **	0.933 **	0.858 **	0.068	0.566 **	−0.004	0.795 **	−0.166															
P_gr._	−0.19	−0.1	0.217	0.174	−0.11	−0.217	−0.633 **	0.183	0.32	0.467 *														
A_ha._	0.357	0.728 **	0.850 **	0.889 **	0.077	0.553 **	0.154	0.805 **	−0.28	0.774 **	−0.001													
P_ha._	−0.490 *	−0.31	−0.194	−0.086	−0.038	−0.353	−0.31	−0.153	0.032	−0.226	−0.06	0.163												
VC	−0.519 *	−0.33	−0.027	−0.14	−0.403	−0.437 *	−0.950 **	0.182	0.909 **	0.078	0.686 **	−0.215	−0.002											
CI_wa._	0.403	0.156	0.23	0.346	0.235	0.104	0.328	0.227	−0.325	0.187	−0.093	0.222	−0.166	−0.291										
CI_wo._	−0.178	−0.051	0.056	0.117	−0.081	−0.142	−0.433 *	0.263	0.424 *	0.1	0.213	0.122	0.109	0.419	−0.065									
CI_gr._	0.11	0.144	0.301	0.238	−0.656 **	0.083	−0.312	0.282	0.271	0.351	0.513 *	0.013	−0.325	0.434 *	−0.076	−0.136								
CI_ha._	0.026	0.258	0.353	0.477 *	−0.015	0.141	0.039	0.397	−0.275	0.29	−0.074	0.616 **	0.620 **	−0.244	0.271	0.213	−0.146							
SI_wa._	0.057	0.003	0.043	0.001	−0.029	−0.004	−0.147	0.081	0.23	0.034	0.193	−0.109	−0.324	0.261	0.544 **	−0.006	0.175	−0.095						
SI_wo._	−0.519 *	−0.327	−0.317	−0.512 *	−0.104	−0.301	−0.329	−0.112	0.561 **	−0.355	−0.095	−0.412	−0.129	0.389	−0.3	−0.019	0.081	−0.316	0.071					
SI_gr._	0.228	0.319	0.523 *	0.42	−0.317	0.23	−0.344	0.422	0.242	0.612 **	0.679 **	0.15	−0.376	0.485 *	0.048	0.021	0.787 **	−0.169	0.228	−0.053				
SI_ha._	0.389	0.18	−0.014	0.209	0.505 *	0.227	0.664 **	−0.157	−0.763 **	−0.031	−0.447 *	0.304	0.255	−0.783 **	0.259	−0.146	−0.624 **	0.341	−0.282	−0.620 **	−0.566 **			
NDVI	−0.480 *	−0.398	−0.083	−0.087	−0.448 *	−0.536 *	−0.850 **	0.22	0.779 **	−0.002	0.532 *	−0.089	0.189	0.832 **	−0.119	0.481 *	0.373	0.03	0.224	0.295	0.26	−0.613 **		
NDBI	−0.920 **	−0.612 **	−0.381	−0.469 *	−0.08	−0.640 **	−0.579 **	−0.293	0.485 *	−0.294	0.248	−0.344	0.389	0.481 *	−0.249	0.076	0.031	−0.055	0.073	0.474 *	−0.127	−0.409	0.436 *	
NDWI	0.645 **	0.447 *	0.089	0.127	0.315	0.581 **	0.916 **	−0.159	−807 **	−0.001	−603 **	0.126	−0.237	−0.885 **	0.16	−0.361	−0.352	0.027	−0.24	−0.394	−0.258	0.608 **	−0.912 **	−0.633 **

** Correlation is significant at the 0.01 level 2-tailed and * Correlation is significant at the 0.05 level 2-tailed. PCI, park cool island intensity; S, size/area; C, circumference; W, width; SI, shape index; A_wa._, area of water bodies; A_la._, area of lawn; A_wo._, area of woodland; A_ha._, area of hard pavement; P_wa._, area proportion of water bodies; P_la._, area proportion of lawn; P_wo._, area proportion of woodland; P_ha._, area proportion of hard pavement; VC, vegetation coverage; CI_wa._, contagion index of water bodies; CI_la._, contagion index of lawn; CI_wo._, contagion index of woodland; CI_ha._, contagion index of hard pavement; SI_wa._, shape index of water dodies; SI_la._, shape index of lawn; SI_wo._, shape index of woodland; SI_ha._, shape index of hard pavement.

## References

[1] Santamouris M. (2015). Analyzing the heat island magnitude and characteristics in one hundred Asian and Australian cities and regions. Sci. Total Environ..

[2] Peng S., Piao S., Ciais P., Friedlingstein P., Ottle C., Bréon F.-M., Nan H., Zhou L., Myneni R.B. (2012). Surface Urban Heat Island Across 419 Global Big Cities. Environ. Sci. Technol..

[3] Kaveh D., Md K., Yan L. (2018). Urban heat island effect: A systematic review of spatio-temporal factors, data, methods, and mit-igation measures. Int. J. Appl. Earth Obs. Geoinf..

[4] Ward K., Lauf S., Kleinschmit B., Endlicher W. (2016). Heat waves and urban heat islands in Europe: A review of relevant drivers. Sci. Total Environ..

[5] Salvati A., Roura H.C., Cecere C. (2017). Assessing the urban heat island and its energy impact on residential buildings in Medi-terranean climate: Barcelona case study. Energy Build..

[6] Skelhorn C., Levermore G., Lindley S. (2016). Impacts on cooling energy consumption due to the UHI and vegetation changes in Manchester, UK. Energy Build..

[7] Kolokotroni M., Zhang Y., Watkins R. (2007). The London Heat Island and building cooling design. Sol. Energy.

[8] Abbassi Y., Ahmadikia H., Baniasadi E. (2020). Prediction of pollution dispersion under urban heat island circulation for different atmospheric stratification. Build. Environ..

[9] Taha H. (1997). Urban climates and heat islands: Albedo, evapotranspiration, and anthropogenic heat. Energy Build..

[10] Li H., Meier F., Lee X., Chakraborty T., Liu J., Schaap M., Sodoudi S. (2018). Interaction between urban heat island and urban pollution island during summer in Berlin. Sci. Total Environ..

[11] Morris K.I., Chan A., Morris K.J.K., Ooi M.C.G., Oozeer M.Y., Abakr Y.A., Nadzir M.S.M., Mohammed I.Y., Al-Qrimli H.F. (2017). Impact of urbanization level on the interactions of urban area, the urban climate, and human thermal comfort. Appl. Geogr..

[12] Lowe S.A. (2016). An energy and mortality impact assessment of the urban heat island in the US. Environ. Impact Assess. Rev..

[13] Yu Z., Guo X., Zeng Y., Koga M., Vejre H. (2018). Variations in land surface temperature and cooling efficiency of green space in rapid urbanization: The case of Fuzhou city, China. Urban For. Urban Green..

[14] Rosenfeld A.H., Akbari H., Bretz S., Fishman B.L., Kurn D.M., Sailor D., Taha H. (1995). Mitigation of urban heat islands: Mate-rials, utility programs, updates. Energy Build..

[15] Rehan R.M. (2016). Cool city as a sustainable example of heat island management case study of the coolest city in the world. HBRC J..

[16] Taleghani M. (2018). Outdoor thermal comfort by different heat mitigation strategies-A review. Renew. Sustain. Energy Rev..

[17] Ali J.M., Marsh S.H., Smith M.J. (2017). A comparison between London and Baghdad surface urban heat islands and possible en-gineering mitigation solutions. Sustain. Cities Soc..

[18] Byrne J., Jinjun Y. (2009). Can urban greenspace combat climate change? Towards a subtropical cities research agenda. Aust. Plan..

[19] Gunawardena K., Wells M., Kershaw T. (2017). Utilising green and bluespace to mitigate urban heat island intensity. Sci. Total Environ..

[20] Völker S., Baumeister H., Clasen T.F., Hornberg C., Kistemann T. (2013). Evidence for the temperature-mitigating capacity of urban blue space—A health geographic perspective. Erdkunde.

[21] Chang C.-R., Li M.-H., Chang S.-D. (2007). A preliminary study on the local cool-island intensity of Taipei city parks. Landsc. Urban Plan..

[22] Steeneveld G., Koopmans S.A., Heusinkveld B.G., Theeuwes N. (2014). Refreshing the role of open water surfaces on mitigating the maximum urban heat island effect. Landsc. Urban Plan..

[23] Sun R., Chen L. (2012). How can urban water bodies be designed for climate adaptation?. Landsc. Urban Plan..

[24] Oliver J.E., Oke T.R. (1979). Boundary Layer Climates. Geogr. Rev..

[25] Qiu K., Jia B. (2020). The roles of landscape both inside the park and the surroundings in park cooling effect. Sustain. Cities Soc..

[26] Wang X., Cheng H., Xi J., Yang G., Zhao Y. (2018). Relationship between Park Composition, Vegetation Characteristics and Cool Island Effect. Sustainability.

[27] Cao X., Onishi A., Chen J., Imura H. (2010). Quantifying the cool island intensity of urban parks using ASTER and IKONOS data. Landsc. Urban Plan..

[28] Cheung P.K., Jim C.Y. (2019). Differential cooling effects of landscape parameters in humid-subtropical urban parks. Landsc. Urban Plan..

[29] Aflaki A., Mirnezhad M., GhaffarianHoseini A., GhaffarianHoseini A., Omrany H., Wang Z.-H., Akbari H. (2017). Urban heat island mitigation strategies: A state-of-the-art review on Kuala Lumpur, Singapore and Hong Kong. Cities.

[30] Hwang Y.H., Lum Q.J.G., Chan Y.K.D. (2015). Micro-scale thermal performance of tropical urban parks in Singapore. Build. Environ..

[31] Lin B.S., Lin C.T. (2016). Preliminary study of the influence of the spatial arrangement of urban parks on local temperature reduc-tion. Urban For. Urban Green..

[32] Lin W., Yu T., Chang X., Wu W., Zhang Y. (2015). Calculating cooling extents of green parks using remote sensing: Method and test. Landsc. Urban Plan..

[33] Feng X., Shi H. (2012). Research on the cooling effect of Xi’an parks in summer based on remote sensing. Acta Ecol. Sin..

[34] Chander G., Markham B. (2003). Revised landsat-5 tm radiometric calibration procedures and post calibration dynamic ranges. IEEE Trans. Geosci. Remote Sens..

[35] Xu H.Q., Shi T.T., Wang M.Y., Fang C.Y., Lin Z.L. (2018). Predicting effect of forthcoming population growth–induced impervi-ous surface increase on regional thermal environment: Xiong’an new area, north China. Build. Environ..

[36] Van De Griend A.A., Owe M. (1993). Determination of microwave vegetation optical depth and single scattering albedo from large scale soil moisture and Nimbus/SMMR satellite observations. Int. J. Remote Sens..

[37] Zhang Y.S., Odeh I.O.A., Han C.F. (2009). Bi-temporal characterization of land surface temperature in relation to impervious sur-face area, NDVI and NDBI, using a sub-pixel image analysis. Int. J. Appl. Earth Obs. Geoinf..

[38] Singh P., Kikon N., Verma P. (2017). Impact of land use change and urbanization on urban heat island in Lucknow city, Central India. A remote sensing based estimate. Sustain. Cities Soc..

[39] Oke T.R., Cermak J.E. (1995). The heat island of the urban boundary layer: Characteristics, causes and effects E. Wind Climate in Cities.

[40] Feyisa G.L., Dons K., Meilby H. (2014). Efficiency of parks in mitigating urban heat island effect: An example from Addis Ababa. Landsc. Urban Plan..

[41] Jaganmohan M., Knapp S., Buchmann C.M., Schwarz N. (2016). The Bigger, the Better? The Influence of Urban Green Space Design on Cooling Effects for Residential Areas. J. Environ. Qual..

[42] Bowler D.E., Buyung-Ali L., Knight T.M., Pullin A.S. (2010). Urban greening to cool towns and cities: A systematic review of the empirical evidence. Landsc. Urban Plan..

[43] Doick K.J., Peace A., Hutchings T.R. (2014). The role of one large greenspace in mitigating London’s nocturnal urban heat island. Sci. Total Environ..

[44] Akbari H. (2009). Cooling Our Communities. A Guidebook on Tree Planting and Lightcolored Surfacing.

[45] Zha Y., Gao J., Ni S. (2010). Use of normalized difference built-up index in automatically mapping urban areas from TM imagery. Int. J. Remote Sens..

[46] Martina P., Luciano M., Giada B., Simone O. (2014). Urban planning indicators: Useful tools to measure the effect of urbanization and vegetation on summer air temperatures. Int. J. Climatol..

[47] Peng J., Xie P., Liu Y., Ma J. (2016). Urban thermal environment dynamics and associated landscape pattern factors: A case study in the Beijing metropolitan region. Remote Sens. Environ..

[48] Zhou W., Wang J., Cadenasso M.L. (2017). Effects of the spatial configuration of trees on urban heat mitigation: A comparative study. Remote Sens. Environ..

[49] Synnefa A., Karlessi T., Gaitani N., Santamouris M., Assimakopoulos D., Papakatsikas C. (2011). Experimental testing of cool colored thin layer asphalt and estimation of its potential to improve the urban microclimate. Build. Environ..

[50] Coutts A.M., Tapper N.J., Beringer J., Loughnan M., Demuzere M. (2012). Watering our cities: The capacity for water sensitive urban design to support urban cooling and improve human thermal comfort in the Australian context. Prog. Phys. Geogr..

